# Rab35 GTPase positively regulates endocytic recycling of cardiac K_ATP_ channels

**DOI:** 10.1080/19336950.2022.2090667

**Published:** 2022-06-27

**Authors:** Bo Yang, Jia-Lu Yao, Jian-Yi Huo, Yu-Long Feng, William A. Coetzee, Guang-Yin Xu, Hua-Qian Yang

**Affiliations:** a Cyrus Tang Medical Institute, Soochow University, Suzhou, Jiangsu, China; b Department of Cardiology, the First Affiliated Hospital of Soochow University, Soochow University, Suzhou, Jiangsu, China; c Department of Cardiology, Dushu Lake Hospital Affiliated to Soochow University, Medical Center of Soochow University, Suzhou Dushu Lake Hospital, Suzhou, Jiangsu, China; d Departments of Pathology, Neuroscience & Physiology, Biochemistry and Molecular Pharmacology, New York University School of Medicine, New York, NY, USA; e Jiangsu Key Laboratory of Neuropsychiatric Diseases, Institute of Neuroscience, Soochow University, Suzhou, Jiangsu, China; f Center for Translational Medicine, The Affiliated Zhangjiagang Hospital of Soochow University, Suzhou, Jiangsu, China

**Keywords:** K_ATP_ channel, Rab35, endocytic recycling, surface density, cardiomyocytes

## Abstract

ATP-sensitive K^+^ (K_ATP_) channel couples membrane excitability to intracellular energy metabolism. Maintaining K_ATP_ channel surface expression is key to normal insulin secretion, blood pressure and cardioprotection. However, the molecular mechanisms regulating K_ATP_ channel internalization and endocytic recycling, which directly affect the surface expression of K_ATP_ channels, are poorly understood. Here we used the cardiac K_ATP_ channel subtype, Kir6.2/SUR2A, and characterized Rab35 GTPase as a key regulator of K_ATP_ channel endocytic recycling. Electrophysiological recordings and surface biotinylation assays showed decreased K_ATP_ channel surface density with co-expression of a dominant negative Rab35 mutant (Rab35-DN), but not other recycling-related Rab GTPases, including Rab4, Rab11a and Rab11b. Immunofluorescence images revealed strong colocalization of Rab35-DN with recycling Kir6.2. Rab35-DN minimized the recycling rate of K_ATP_ channels. Rab35 also regulated K_ATP_ channel current amplitude in isolated adult cardiomyocytes by affecting its surface expression but not channel properties, which validated its physiologic relevance and the potential of pharmacologic target for treating the diseases with K_ATP_ channel trafficking defects.

## GRAPHICAL ABSTRACT

**Figure:**
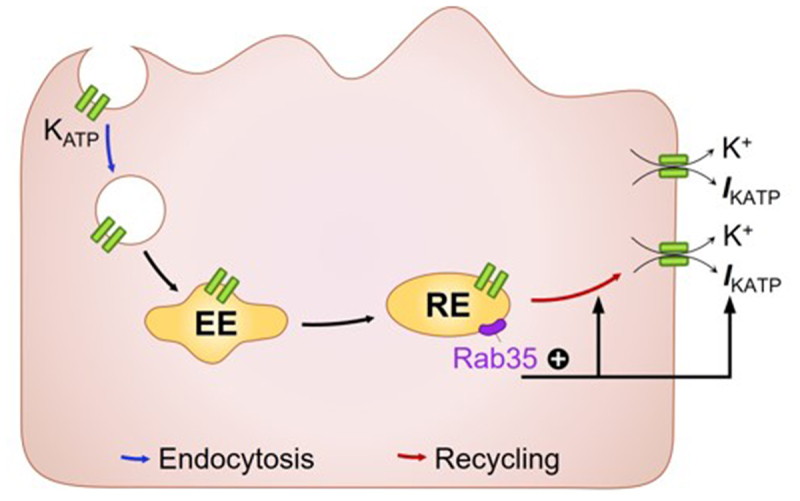

## Introduction

Sarcolemmal ATP-sensitive K^+^ (K_ATP_) channels uniquely couple intracellular energy metabolism to cell membrane excitability, regulating diverse physiologic processes including insulin secreting, blood flow, neurotransmitter release and action potential duration adaptation [1,2]. Decreased K_ATP_ channel surface expression caused by genetic variations or pathophysiologic conditions are associated with hypoglycemia, diabetes mellitus, atrial fibrillation, dilated cardiomyopathy and cardiac ischemia [1]. Therefore, strategies to maintain K_ATP_ channel surface density have a high therapeutic potential.

K_ATP_ channel is composed of four pore-forming inward rectifier potassium channel (Kir6) subunits and four regulatory sulfonylurea receptor (SUR) subunits. Both Kir6 and SUR subunits have the ER retention motif (RKR), and the formation of K_ATP_ channel octamer has been proposed to shield this motif to facilitate ER exit [3]. Further, SUR subunits are glycosylated in the Golgi apparatus before reaching the cell surface [4,5]. Several scaffolding proteins, including ankyrins, have been shown to anchor the K_ATP_ channels on cell membrane [6–8]. Internalization of K_ATP_ channels occurs mainly through clathrin-coated vesicles [9]. Experiments with antibody capture assays have demonstrated that K_ATP_ channels can be recycled, which occurs at a time scale of 10–15 min [10]. However, the molecular mechanism of K_ATP_ channel recycling is poorly understood.

Rab family members serve as multifaceted organizers of almost all membrane trafficking processes by consuming GTP in eukaryotic cells. More than 60 Rab protein family members are distributed in different intracellular membranes, controlling multiple processes such as the generation, movement, fusion, and recruitment of intracellular vesicles that transport membrane proteins. Within the Rab family, Rab4 mediates fast endocytic recycling directly from early endosomes, whereas Rab11a, Rab11b and Rab35 mediate slow endocytic recycling through recycling endosomes [11,12].

The aim of this study is to investigate the involvement of Rab GTPases in K_ATP_ channel endocytic recycling, and our data demonstrate that, of the four recycling-related Rab GTPases, only Rab35 promotes K_ATP_ channel endocytic recycling and increases its surface expression in both heterologous cell system and cardiomyocytes.

## Materials and methods

### cDNA constructs

Plasmids of Kir6.2 and SUR2A without tags are kindly supplied by Dr. Lei Chen (Peking University, Beijing, China) [13]. Dominant negative Rab constructs (GFP-Rab4-S22N, GFP-Rab11a-S25N, GFP-Rab11b-S25N, GFP-Rab35-S22N) and Avi-Kir6.2–4HA plasmids were synthesized by Genscript. Constitutively active Rab35 (GFP-Rab35-Q67L) plasmid were synthesized by HanBio. HA-Kir6.2 was previously used [14].

### Inside-out K_ATP_ current recording

Inside-out patch clamping was performed by using an Axopatch 200B amplifier, and data were recorded with a Digidata 1550B and Clampex 11 software, digitized at 5 kHz. The cytosolic ATP concentration was changed by using MappingLab PVG-08. The resistance of the pipette was 4 ~ 5 MΩ when filled with pipette solution (in mM: 30 KCl, 2 CaCl_2_, 1 MgCl_2_, 10 HEPES, 110 potassium gluconate, and pH 7.4). ATP was dissolved in base solution (in mM: 30 KCl, 1 EGTA, 1 MgCl_2_, 10 HEPES, 110 potassium gluconate, and pH 7.2). After patch excision, pipette potential was held at +80 mV and currents were recorded immediately to minimize current rundown. Current traces were analyzed by Clampfit 11 for open probability and single channel current. Recordings in the absence of ATP for 10 seconds were filtered at 1 kHz and subjected to all-point histograms, which were further fitted with Gaussian distributions. The parameters (A, μ, σ) generated from Gaussian fitting were used to calculate open probability and single channel current.

### Biotinylation assay

HEK293 cells transfected with Avi-Kir6.2–4HA/SUR2A (PolyJet™ In Vitro DNA Transfection Reagent, SL100688, SignaGen Laboratories) were incubated at 4°C for 1 h with 0.33 mg/ml biotin in PBS buffer, and reaction was terminated by quenching solution. After washing twice with TBS buffer, cell lysates were prepared in RIPA buffer containing 1% protease inhibitor cocktail, 0.2% PMSF and 5 mM EDTA. Lysates were mixed with Neutravidin Agarose Beads (89881, Thermo Fisher Scientific), and rotated by the shaker at 4°C overnight. Centrifugated at 1000 g for 1 min, supernatants were discarded, and agarose beads were washed three times with PBS, and then mixed with 4x Laemmli Protein Sample Buffer (1610747, Biorad) containing 50 mM DTT for 1 h at room temperature to elute biotinylated proteins.

### Western blotting

Total cell lysates and biotinylated proteins were subjected to SDS-PAGE electrophoresis. The primary antibodies used include mouse anti-HA (901501, 1:5000, BioLegend), mouse anti-GAPDH (FD0063, 1:10,000, FuDeBio), mouse anti-Akt (sc-5298, 1:2000, Santa Cruz Biotechnology), rabbit anti-p-Akt (4060, 1:2000, Cell Signaling Technology). The secondary antibodies used were IRDye® 800CW Goat anti-Mouse IgG Secondary Antibody (926–32210, LI-COR Biosciences), IRDye® 680RD Goat anti-Rabbit IgG Secondary Antibody (926–68071, LI-COR Biosciences). LY294002 (L832989, 10 μM, Macklin) was applied for 24 h to inhibit PI3K signaling.

### Immunofluorescence

Hela cells transfected with HA-Kir6.2/SUR2A and GFP-tagged dominant negative Rab GTPases were incubated overnight with mouse anti-HA antibody (901501, 1:200 dilution in DMEM containing 10% donkey serum, BioLegend). Cells were fixed with 4% paraformaldehyde in PBS for 15 min, permeabilized by 1% Triton X-100 for 15 min, and blocked with 5% donkey serum in PBS for 30 min. Cy™3 AffiniPure Donkey anti-mouse IgG (715–165-151, 1: 500, Jackson ImmunoResearch Laboratories) was diluted in blocking solution, and incubated at room temperature in dark room for 45 min. After washing with PBS three times, cells were mounted with DAPI Fluoromount-G (0100–20, SouthernBiotech). Images were collected by Olympus FV3000 confocal Microscope.

For recycling double staining assay, after overnight HA antibody incubation, the cells were washed with DMEM and surface-bound antibodies were labeled at room temperature for 0.5 h by Alexa Fluor647 AffiniPure Donkey anti-mouse IgG (715–605-151, 1: 500, Jackson ImmunoResearch Laboratories). The cells were then incubated at 37°C for 2 h to allow recycling of internalized channels back to cell surface. The cells were then fixed with 4% paraformaldehyde and HA-antibody marked channels recycled back to the cell surface were labeled with Cy™3 AffiniPure Donkey anti-mouse IgG (715–165-151, 1: 500, Jackson ImmunoResearch Laboratories) and non-recycled channels were labeled with Alexa Fluor647 AffiniPure Donkey Anti-mouse IgG (715–605-151, 1: 500, Jackson ImmunoResearch Laboratories) after permeabilization.

### Isolation and infection of adult rat ventricular cardiomyocytes

Ventricular cardiomyocytes were isolated from male Sprague-Dawley rats (9–11 weeks old, purchased from Vital River). Hearts were rapidly excised after anesthesia and rinsed with ice-cold Tyrode’s solution (in mM: 137 NaCl, 5.4 KCl, 10 HEPES, 1 MgCl_2_, 0.33 NaH_2_PO_4_, 1.8 CaCl_2_, 10 glucose, and pH 7.4). The hearts were cannulated and perfused in Langendorff mode with Ca^2+^-free Tyrode’s solution for 2 min. The perfusate was then switched to Ca^2+^-free Tyrode’s solution containing 1.56 mg/ml collagenase type II (LS004176, Worthington) for 20 min. The enzyme was washed out by 2 min perfusion with KB solution (in mM: 20 taurine, 50 L-glutamic acid, 10 HEPES, 10 Glucose, 1 EGTA, 3 MgCl_2_, 20 KH_2_PO_4_, 40 KCl, and pH 7.2). Hearts were removed from the cannula and cut into pieces and the supernatant was kept after resuspending by KB solution followed by MEM medium (PM150410, Procell) containing 1% penicillin-streptomycin. Cells were plated on laminin (L2020, 20 µg/ml, Sigma)-coated coverslips and cultured in MEM medium. Adenoviruses that carry Rab35-S22N or Rab35-Q67L (purchased from HanBio) were added at a multiplicity of infection of 1000 for a 24 h incubation. Infected cardiomyocytes were labeled by mCherry expression. Cultured cardiomyocytes were used for patch at 48 h post-infection. The animal study protocol was approved by the Ethics Committee of Soochow University (SUDA20210930A03).

### Reverse transcription – polymerase chain reaction

Rab35 expression in HEK293 and rat heart were analyzed by reverse transcription – polymerase chain reaction. Glyceraldehyde 3-phosphate dehydrogenase (GAPDH) expression was analyzed as control. Total RNAs were isolated from HEK293 cells and rat heart using Trizol reagents (Invitrogen, 15596018) to make first-strand cDNAs with the HiScript III 1st Strand cDNA Synthesis kit (Vazyme, R312-01). PCR amplification of Rab35 transcripts was done using forward (5’- AAGCTGCAGATCTGGGACAC −3’) and reverse (5’- CGTCGTAAACCACAATGACCC −3’) primers in 30 cycles of 15s denaturation at 95°C, 20s annealing at 60°C, and 30s elongation at 72°C. As control, primers for GAPDH (forward primer 5’- CCTGCACCACCAACTGCTTA −3’, reverse primer 5’- AGTGATGGCATGGACTGTGG −3’) were included in parallel reactions. Both Rab35 and GAPDH primers were designed to fully match human and rat sequences. Amplified PCR products were examined by agarose gel electrophoresis.

### Statistical analysis

Western blotting gray value and Manders coefficient were obtained with Image J and python scripts. Data processing of patch traces were analyzed by Clampfit 11. When comparing two groups, the Student’s t-test is applied. One-way or two-way ANOVA was used for comparison of multiple groups, followed by the Holm-Sidak’s analysis. Values of *P* < 0.05 were considered significant.

## Results

### Dominant negative Rab35 downregulates K_ATP_ channel current amplitude

**Figure 1.**
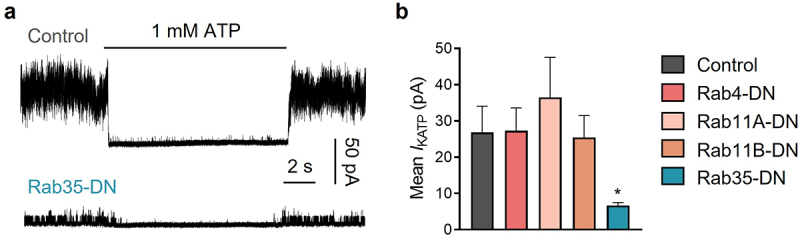
Of the four Rab GTPases related to endocytic recycling, only Rab35-DN (dominant negative mutant) reduces K_ATP_ current amplitude. (a) Representative inside-out current traces in control and Rab35-DN groups recorded from HEK293 cells transfected with Kir6.2/SUR2A. The presence of 1 mM ATP is indicated. (b) Summary of mean K_ATP_ currents of all control and four dominant negative Rab GTPases (Rab4-DN, Rab11a-DN, Rab11b-DN, Rab35-DN). n ≥ 18 current traces in each group. *P < 0.05 vs. the control group determined by one-way ANOVA followed by the Holm-Sidak’s analysis.

To examine the involvement of Rab GTPases in K_ATP_ channel endocytic recycling, we individually co-expressed GDP-locked dominant-negative (DN) mutants of Rab4, Rab11a, Rab11b and Rab35 [15], with K_ATP_ channel subunits in HEK293 cells. Each of the Rab GTPase mutants were concatenated with EGFP at the N-terminus. We measured K_ATP_ channel currents with inside-out membrane patches in ATP-free solutions. K_ATP_ channel mean patch current was significantly decreased in cells expressing dominant-negative Rab35 mutant (Rab35-DN), whereas Rab4-DN, Rab11a-DN and Rab11b-DN had no effect on K_ATP_ channel current amplitude (Figure 1).

### Rab35 does not affect K_ATP_ channel intrinsic properties

**Figure 2.**
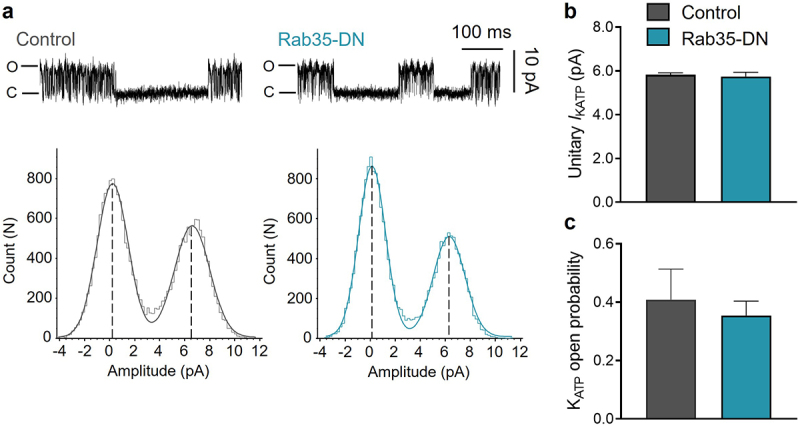
Rab35-DN does not affect K_ATP_ channel unitary current and open probability. (a) Upper panels, representative K_ATP_ channel inside-out single channel recordings in control and Rab35-DN groups; lower panels, all-points histograms constructed from events in current traces and fitted to Gaussian distribution curves. (b) Summary of K_ATP_ channel unitary current amplitude and (c) open probability in control and Rab35-DN groups. n ≥ 11 current traces in each group.

We next determined whether Rab35-DN has a direct effect on K_ATP_ channel opening. Using the inside-out patch clamp configuration, we determined the unitary current amplitude of K_ATP_ channels using all-point histograms and curve fitting to Gaussian distributions (Figure 2(a)). The unitary K_ATP_ channel current and open probability were 5.82 ± 0.08 pA and 0.41 ± 0.10 for control, and were unaffected by co-expression with Rab35-DN, with 5.74 ± 0.19 pA and 0.35 ± 0.05 respectively (Figure 2(b,c)). The K_ATP_ channel unitary current and open probability co-expressed with Rab4-DN, Rab11a-DN or Rab11b-DN were also unchanged (Supplementary Figure S1).

### Dominant-negative Rab35 decreases K_ATP_ channel surface density

**Figure 3.**
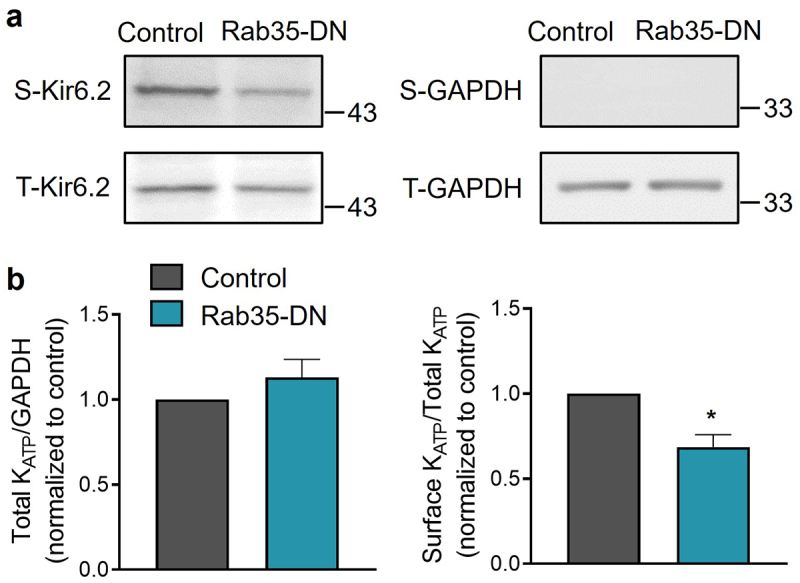
Rab35-DN reduces K_ATP_ channel surface density. (a) HEK293 cells transfected with Avi-Kir6.2–4HA/SUR2A were surface biotinylated, and western blotting was performed with anti-HA and anti-GAPDH Abs. Representative blots of total (t) and biotinylated surface (s) Kir6.2 and GAPDH are shown. (b) Ratios of total Kir6.2 to total GAPDH and surface Kir6.2 to total Kir6.2 are shown for control and Rab35-DN groups. n ≥ 10 blots/group. *P < 0.05 vs. the control group with student’s t-test.

Electrophysiological data are consistent with the concept that Rab35-DN impairs K_ATP_ channel surface expression. We tested this possibility biochemically using an engineered Kir6.2 expressing an extracellular Avi tag and C-terminal HA tags (Avi-Kir6.2–4HA). Avi-Kir6.2–4HA/SUR2A channels were expressed in HEK293 cells. Surface proteins were purified using a surface biotinylation assay, and Avi-Kir6.2–4HA was detected with immunoblotting. We found that co-expression of Rab35-DN with Avi-Kir6.2–4HA/SUR2A did not affect the total amount of Kir6.2, but significantly decreased its surface expression (Figure 3). The total and surface expression of K_ATP_ channels co-expressed with Rab4-DN, Rab11a-DN and Rab11b-DN were also investigated and found to be unchanged (Supplementary Figure S2). Both the electrophysiological and biochemical data demonstrate that dominant-negative Rab35, but not Rab4, Rab11a or Rab11b, downregulates K_ATP_ channel surface density.

### Dominant-negative Rab35 retains recycled K_ATP_ channels in intracellular vesicles

**Figure 4.**
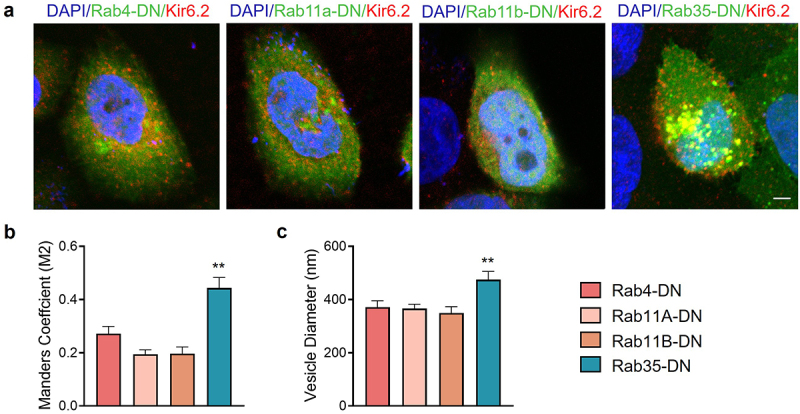
Rab35-DN induces intracellular accumulation of K_ATP_ channels. (a) Representative immunostaining images obtained from Hela cells transfected with HA-Kir6.2/SUR2A (red) and GFP-tagged Rab4-DN, Rab11a-DN, Rab11b-DN or Rab35-DN (green). (b) Manders M2 colocalization coefficient was calculated as the presence of HA-Kir6.2 in the GFP-labeled vesicles. (c) Average diameters of the vesicles containing K_ATP_ channels compared in Rab4-DN, Rab11a-DN, Rab11b-DN and Rab35-DN groups. n ≥ 11 images in each group. Scale bar, 5 µm. **P < 0.01 determined by one-way ANOVA followed by Holm-Sidak test.

To investigate the location and trafficking of recycled K_ATP_ channels, we used a Kir6.2 subunit with an HA tag in its extracellular loop (HA-Kir6.2) to label only recycled K_ATP_ channels but not newly synthesized channels. After an incubation for 2 h with HA antibody, surface and recycling K_ATP_ channels were labeled. We then performed an immunofluorescence experiment to test the colocalization of recycling K_ATP_ channels with dominant negative Rab GTPases (Figure 4(a)). Statistically, recycling K_ATP_ channels had a significantly higher colocalization coefficient with Rab35-DN compared to Rab4-DN, Rab11a-DN and Rab11b-DN (Figure 4(b)), indicating that K_ATP_ channels recycled through Rab35-drived compartments, and Rab35-DN induced accumulation of K_ATP_ channels in these intracellular vesicles, shown by enlarged vesicle size (Figure 4(c) and Supplementary Figure S3).

### K_ATP_ channel recycling is minimized by dominant negative Rab35

**Figure 5.**
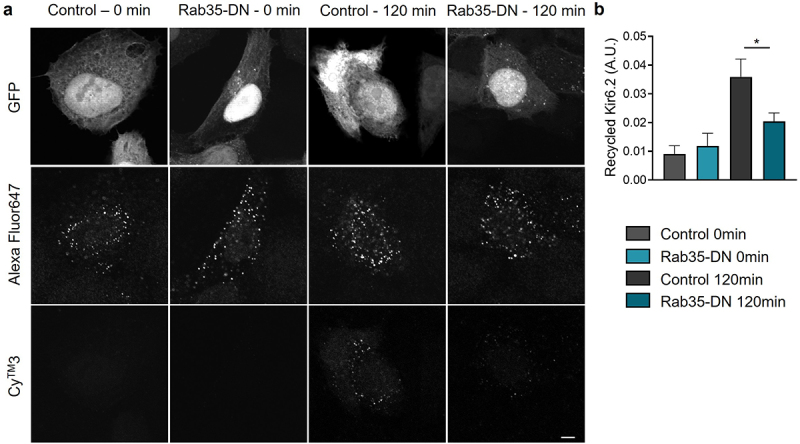
K_ATP_ channel recycling is minimized by Rab35-DN. (a) Representative immunostaining images obtained from Hela cells transfected with HA-Kir6.2/SUR2A and GFP-tagged Rab35-DN. Recycled HA-Kir6.2 was labeled with Cy^TM^3-conjugated secondary antibody. The total amount of K_ATP_ channels in the process of endocytosis and recycling was labeled with Alexa Fluor647-conjugated secondary antibody. Scale bar, 5 µm. (b) Quantification of the recycled HA-Kir6.2 as the ratio of Cy^TM^3 fluorescence intensity to Alexa Fluor647 fluorescence intensity in control and Rab35-DN groups for recycling time courses of 0 min and 120 min. n ≥ 5 images in each group. *P < 0.05 determined by two-way ANOVA followed by Holm-Sidak test.

To directly investigate the recycling process of K_ATP_ channels, we introduced a double staining assay [16] using Kir6.2 with an extracellular HA tag (HA-Kir6.2). HA antibody incubation labeled the K_ATP_ channels in the process of endocytosis and recycling. K_ATP_ channels recycled back to the cell membrane were labeled with Cy™3-conjugated secondary antibody, while the K_ATP_ channel pool for recycling were labeled with Alexa Fluor 647-conjugated secondary antibody. The ratio of Cy^TM^3 to Alexa Fluor647 fluorescence intensity indicated the K_ATP_ channel recycling rate. As shown in Figure 5(a), at t = 0 min, no recycled K_ATP_ channels were detected, while at t = 120 min, a significant amount of Cy™3 labeled channels were observed. When co-expressed with Rab35-DN, the amount of K_ATP_ channels recycled back to the cell membrane was dramatically reduced (Figure 5(b)), indicating a direct involvement of Rab35 in K_ATP_ channel recycling.

### Rab35 regulates K_ATP_ channel recycling independent of PI3K signaling

**Figure 6.**
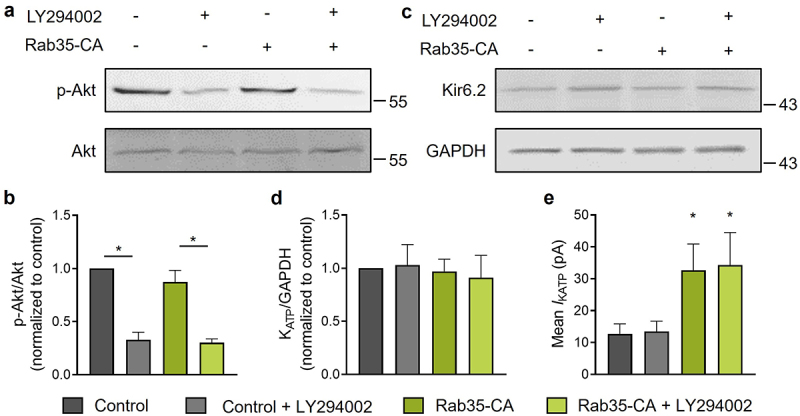
Rab35 regulates K_ATP_ channel recycling independent of PI3K signaling. (a) HEK293 cells expressing Avi-Kir6.2–4HA/SUR2A transfected with or without Rab35-CA (constitutively active Rab35 mutant) were treated with or without 10 μM LY294002 (PI3K inhibitor) for 24 h. Representative blots of total and phosphorylated Akt and (b) ratios of phosphorylated Akt to total Akt were shown for indicated groups (n = 5 blots). (c) Representative blots of Kir6.2 and GAPDH and (d) ratios of Kir6.2 to GAPDH were shown for indicated groups (n = 5 blots). (e) Summary of mean K_ATP_ channel currents of indicated groups. n ≥ 13 current traces in each group. *P < 0.05 vs. the control group determined by two-way ANOVA followed by Holm-Sidak test.

Rab35 directly binds to and controls the activity of p85α, the regulatory subunit of PI3K [17]. Several studies showed that Rab35 may act as an upstream activator of PI3K/Akt signaling [17,18] and PI3K activation has been shown to increase K_ATP_ channel surface density in neurons and pancreatic β-cells [19,20]. To test the possibility of the involvement of PI3K in Rab35-mediated K_ATP_ channel recycling, we used the PI3K inhibitor LY294002 to impair PI3K activation, which decreased Akt phosphorylation (Figure 6(a,b)). Without changing the total K_ATP_ channel expression (Figure 6(c,d)), a constitutively active Rab35 (Rab35-CA) significantly increased K_ATP_ channel mean patch current (Figure 6(e)) and the current amplitudes in both control and Rab35-CA groups were unaffected by PI3K inhibitor LY294002 (Figure 6(e)), demonstrating that Rab35 regulates K_ATP_ channel recycling independent of PI3K signaling.

### Active Rab35 enhances K_ATP_ channel surface density in cardiomyocytes

**Figure 7.**
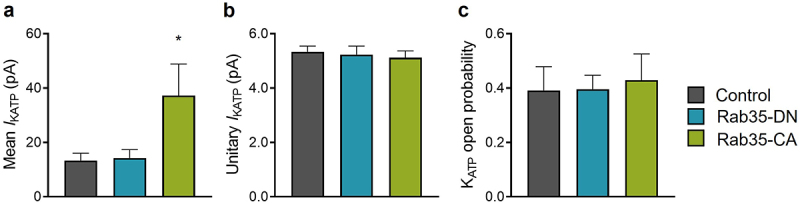
Rab35 regulates native K_ATP_ channels in ventricular cardiomyocytes. (a) Summary of mean K_ATP_ current amplitudes, (b) K_ATP_ channel unitary current and (c) open probability of control, Rab35-DN and Rab35-CA groups. n ≥ 5 in each group. *P < 0.05 vs. the control group determined by one-way ANOVA followed by the Holm-Sidak’s analysis.

To elucidate the physiological relevance of the regulation of K_ATP_ channel recycling by Rab35, we studied the functional role of Rab35 in isolated rat adult cardiomyocytes by adenoviral delivery of Rab35-DN, Rab35-CA, or mCherry as a control. Consistent with the observations in transfected HEK293 cells, K_ATP_ channel mean patch current was significantly increased by Rab35-CA (Figure 7(a)) without changing intrinsic K_ATP_ channel properties (Figure 7(b,c)). However, Rab35-DN was without effect on K_ATP_ channel current amplitude in rat cardiomyocytes (Figure 7(a)). Several databases, including The Human Protein Atlas and Proteomics DB, show a low expression of Rab35 in the heart among different tissues, and we confirmed that the Rab35 mRNA expression level was significantly lower in rat heart compared to HEK293 cells (Supplementary Figure S4), indicating that Rab35-DN could not decrease K_ATP_ channel current in rat cardiomyocytes due to the low expression of endogenous Rab35.

## Discussion

Several Rab GTPases have been reported to be directly involved in the trafficking of ion channels in cardiomyocytes, including Rab4, Rab5, Rab7, Rab9, and Rab11 [21]. A novel finding of this study is the identification of K_ATP_ channel as the first ion channel in cardiomyocytes recycled in a Rab35-dependent manner.

Rab35 contains an evolutionarily conserved polybasic C-terminal tail and localizes both at the intracellular vesicles and plasma membrane [22,23]. Rab35 plays an important role in promoting endocytic recycling of various cargoes, including Ca^2+^-activated K^+^ channel KCa2.3 in terms of ion channels. Rab35-DN, used in this work as well, resulted in accumulation of KCa2.3 in an intracellular compartment and a decrease in steady-state plasma membrane expression in endothelial cells [24]. However, KCa3.1 recycled in a Rab35-independent manner [24]. Rab4 or Rab11 does not affect K_ATP_ channel trafficking in our study but regulates Kv1.5 recycling in atrial myocytes [16], indicating the cargo specificity of Rab GTPases in mediating intracellular trafficking of ion channels.

The number of surface K_ATP_ channels varies dynamically, and the increase of surface K_ATP_ channels can occur within minutes [25], indicating that this regulation is not at the transcription or translation level, but through recruitment of stored K_ATP_ channels in intracellular vesicles to traffic to cell surface. Rab4 mediates fast endocytic recycling directly from early endosomes, whereas Rab11 and Rab35 mediate slow endocytic recycling through recycling endosomes. K_ATP_ channel recycling occurs at a time scale of 10–15 min [10], indicating the involvement of slow endocytic recycling. Indeed, we observed colocalization of endocytosed K_ATP_ channels with Rab35-DN positive vesicles (Figure 4) and Rab35-DN decreased the recycling rate of K_ATP_ channels (Figure 5), demonstrating that Rab35 is a key mediator of K_ATP_ channel recycling. We previously reported that Rab11a was also colocalized with K_ATP_ channels, and regulated K_ATP_ channel surface expression [14]. However, the colocalization experiment performed in the previous study was between Rab11a and total K_ATP_ channels while in the present study between Rab35 and recycling K_ATP_ channels. Moreover, based on the calculation of current amplitude difference, the amount of expressed K_ATP_ channels in the previous experiment with Rab11a was ~100 fold higher than that with Rab35 in the present study, which indicates that Rab35 may be the primary regulator of K_ATP_ channel recycling, while overexpressed K_ATP_ channel may utilize Rab11a-containing vesicles for trafficking as well.

In the regulation of K_ATP_ channel trafficking, another Rab GTPase, Rab8a, has also been shown to affect K_ATP_ channel surface density [26]. Rab8a colocalized and co-immunoprecipitated with Kir6.2 subunit and knockdown of Rab8a reduced K_ATP_ channel surface expression [26]. Rab8a is not a marker protein for ERC, but can be recruited to ERC by Rab35 through the interaction with molecule interacting with CasL-like protein 1 (MICAL)-L1, which is a Rab35 effector [27,28], indicating that Rab8a may be a cooperator of Rab35 in regulating K_ATP_ channel recycling. The Rab35/MICAL-L1 complex can recruit not only Rab8a, but also Rab13, Rab36 and Eps15 homology domain protein family 1 (EHD1) [27,29,30]. However, a dominant negative EHD1 construct was reported to have no effect on K_ATP_ channel recycling and surface density [14,31], suggesting that not all Rab35 effectors contribute to regulate K_ATP_ channel recycling and whether Rab13 or Rab36 affect K_ATP_ channel recycling needs further investigation. It is well known that leptin promotes K_ATP_ channel recycling via AMPK and PI3K pathways [20,25], and ischemia preconditioning maintains K_ATP_ channel surface density to protect cardiomyocytes against ischemic injury in an AMPK-dependent and PKC-dependent manner [32,33]. AMPK, PI3K and PKC are all phosphorylation kinases and Rab35 has been shown to be phosphorylated at Thr72 by LRRK2 in the process of Parkinson’s disease [34]. Whether Rab35 phosphorylation is involved in regulating K_ATP_ channel recycling and cardioprotection needs further investigation.

We and others have shown that K_ATP_ channels at the surface membrane mediate the protective effect of ischemic preconditioning [2,32,35]. Maintaining and increasing K_ATP_ channel surface density protect cardiomyocytes against ischemic injury [14]. Constitutively active Rab35 mutant increased endogenous K_ATP_ channel current, indicating that stimulating Rab35 may represent as a future therapy for treating ischemic cardiac injury. Indeed, it is well known that exercise is protective to the heart, partially mediated by surface K_ATP_ channels, and in an exercised mouse model, swimming increased the expression of Rab35 at both the mRNA and protein levels [36]. The role of the link between Rab35 and K_ATP_ channel in cardioprotection needs to be further investigated.

## Conclusion

This study demonstrated that Rab35 is the key regulator of K_ATP_ channel endocytic recycling. Inactivation of Rab35 downregulates K_ATP_ channel current amplitude by reducing K_ATP_ channel surface expression without affecting channel properties. Activation of Rab35 in cardiomyocytes upregulates K_ATP_ channel current, indicating Rab35 as the potential pharmacologic target for treating the diseases due to K_ATP_ channel trafficking defects.

## Supplementary Material

Supplemental Material

## Data Availability

The data that support the findings of this study are available from the corresponding author upon reasonable request.https://doi.org/10.5061/dryad.w6m905qs0
