# Supplementary material for: Rab35 GTPase positively regulates endocytic recycling of cardiac KATP channels
Source: Channels (Austin). 2022 Jun 27;16(1):137–47. doi: 10.1080/19336950.2022.2090667 (PMC9721419; doi:10.1080/19336950.2022.2090667)
Supplement: Supplemental Material [file KCHL_A_2090667_SM3873.docx]

**Supplementary Materials**

**Rab35 GTPase positively regulates endocytic recycling of cardiac K_ATP_ channels**

Bo Yang ^a^, Jia-Lu Yao ^b,c^, Jian-Yi Huo ^a^, Yu-Long Feng ^a^, William A. Coetzee ^d^, Guang-Yin Xu ^e,f,*^ and Hua-Qian Yang ^a,*^

**
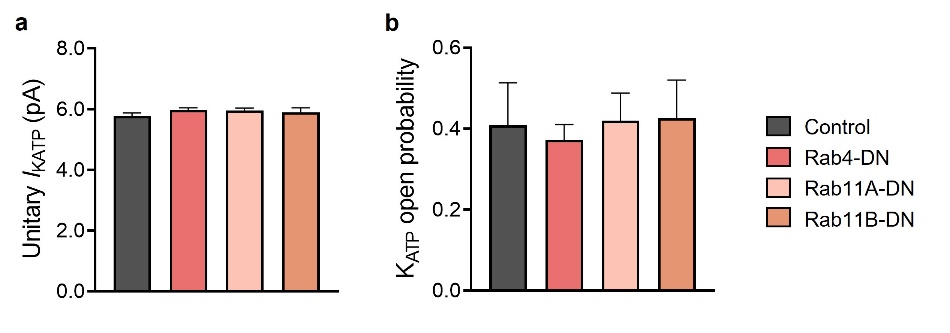
**


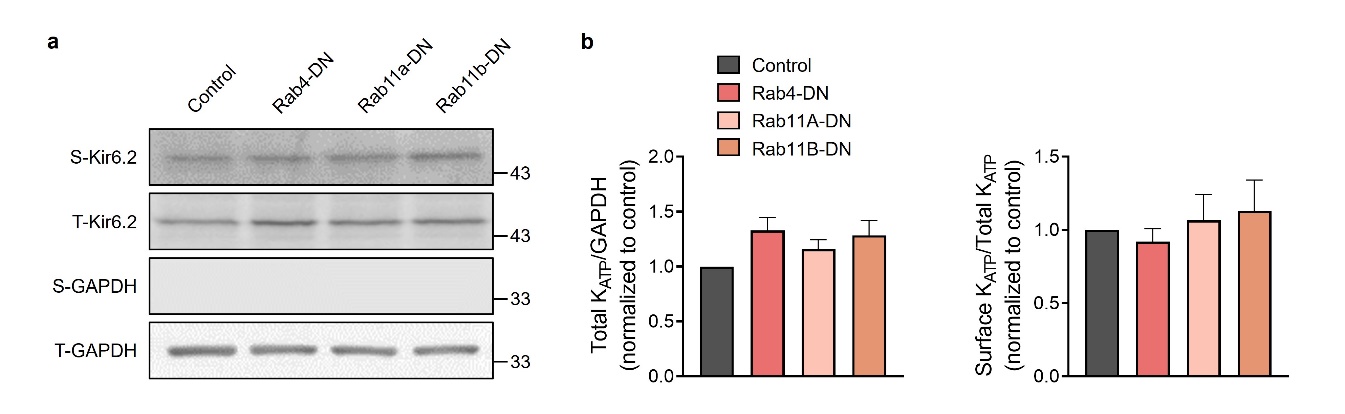
**Supplementary Figure S1.** Rab4, Rab11a and Rab11b do not affect K_ATP_ channel unitary current and channel open probability. Summary of (a) K_ATP_ channel unitary current and (b) open probability in control, Rab4-DN, Rab11a-DN and Rab11b-DN groups. n ≥18 current traces in each group.

**Supplementary** **Figure S2.** Rab4, Rab11a and Rab11b do not affect K_ATP_ channel surface density. HEK293 cells transfected with Avi-Kir6.2-4HA/SUR2A were surface biotinylated, and neutravidin beads were used to enrich biotinylated proteins, and then western blotting was performed with anti-HA and anti-GAPDH antibodies. (a) Representative blots of total (T) and biotinylated surface (S) Kir6.2 and GAPDH are shown. (b) Ratios of total Kir6.2 to total GAPDH and surface Kir6.2 to total Kir6.2 are shown for control, Rab4-DN, Rab11a-DN and Rab11b-DN groups. n ≥ 9 blots/group.


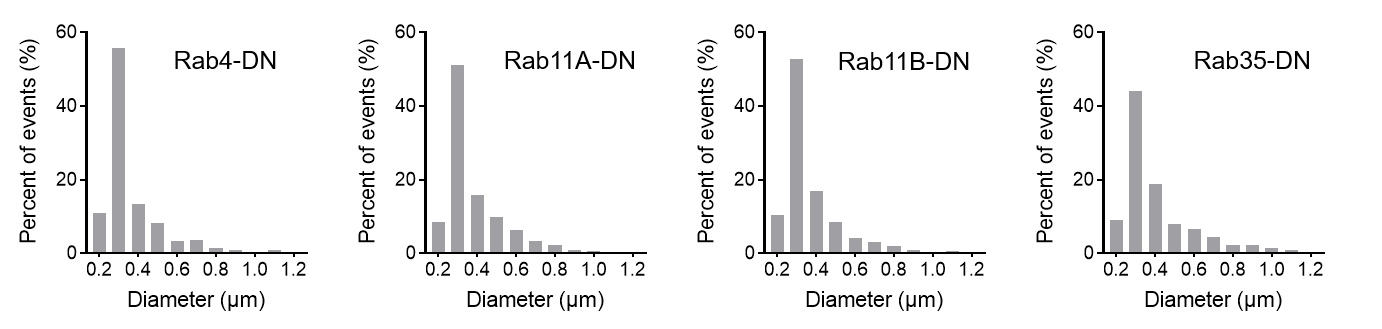


**Supplementary Figure S3.** Histogram distributions of the diameters of vesicles containing recycled K_ATP_ channels in Rab4-DN, Rab11a-DN, Rab11b-DN and Rab35-DN groups.

**
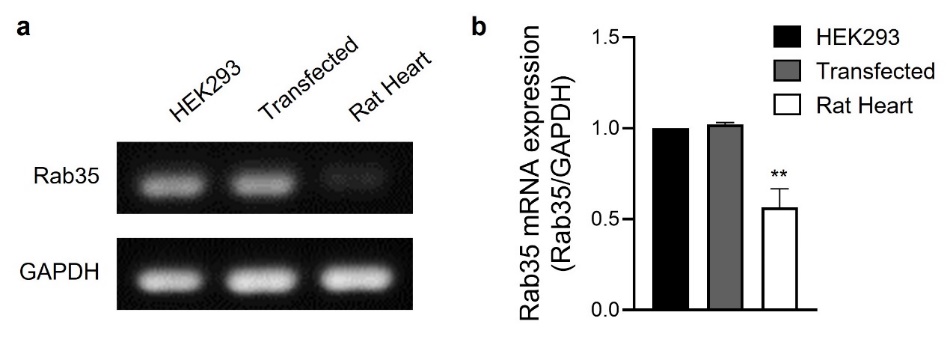
**

**Supplementary Figure S4.** Rab35 mRNA expression is lower in rat heart compared to HEK293 cells. (a) Representative agarose gel images and (b) ratios of Rab35 to GAPDH expression in HEK293 cells, HEK293 with transfection of K_ATP_ channel subunits and rat heart. ***P* < 0.01 vs. the HEK293 group determined by one-way ANOVA followed by the Holm-Sidak’s analysis. n = 3 in each group.
